# Pseudouridine Synthase 7 in Cancer: Functions, Mechanisms, and Therapeutic Potential

**DOI:** 10.3390/cells14171380

**Published:** 2025-09-04

**Authors:** Qiwei Yang, Thomas G. Boyer, Ayman Al-Hendy

**Affiliations:** 1 Department of Obstetrics and Gynecology, University of Chicago, Chicago, IL 60637, USA; aalhendy@bsd.uchicago.edu; 2 Department of Molecular Medicine, UT Health San Antonio, San Antonio, TX 78229, USA; boyer@uthscsa.edu; 3 Department of Medical Sciences, Khalifa University, Abu Dhabi P.O. Box 127788, United Arab Emirates

**Keywords:** PUS7, RNA modification, pseudouridine synthases, pseudouridylation, epitranscriptomic remodeling, cancer, translational reprogramming, stress adaptation, cofactors, pathways, cell proliferation, invasion, tumorigenicity, PUS7 inhibitor, RNA-based targeted therapy, gynecological disorder

## Abstract

Pseudouridylation, the most abundant RNA modification, plays a critical role in modulating RNA structure, stability, and function. Among the family of pseudouridine synthases, Pseudouridine Synthase 7 (PUS7) has recently gained attention for its emerging roles in human health and disease. Originally characterized for its function in modifying tRNA and small non-coding RNAs, PUS7 is now recognized as a dynamic regulator of mRNA pseudouridylation, influencing gene expression at the post-transcriptional level. Aberrant expressions or activity of PUS7 have been linked to a variety of pathological conditions, including cancers such as colon cancer, glioblastoma, pancreatic cancer, and neuroblastoma, as well as potential roles in neurodevelopmental disorders and immune regulation. Through mechanisms involving translational reprogramming, stress adaptation, and epitranscriptomic remodeling, PUS7 contributes to disease progression and cellular plasticity. This review summarizes the current understanding of PUS7 biology, its functional relevance in the contexts of cancer progression, and the growing interest in targeting RNA-modifying enzymes for therapeutic intervention. Uncovering the full spectrum of PUS7-mediated pseudouridylation and its downstream effects holds promise for advancing our understanding of RNA-based regulation in human diseases, including gynecological disorders.

## 1. Introduction

### 1.1. Importance of Post-Transcriptional RNA Modifications in Gene Regulation

Post-transcriptional RNA modifications have emerged as crucial regulators of gene expression, adding an additional layer of control beyond the DNA sequence [1,2,3,4]. These chemical modifications, such as methylation (e.g., m^6^A, m^5^C), pseudouridylation, acetylation, and others, can influence RNA stability, splicing, export, localization, and translation. By dynamically modifying RNA molecules in response to developmental cues or environmental stressors [5], cells can rapidly fine-tune gene expression without altering the underlying genome [2,3,6,7,8]. For instance, modifications like N^6^-methyladenosine (m^6^A) can recruit specific binding proteins to modulate RNA decay or translation [7,9,10,11,12,13], while pseudouridylation can enhance RNA structural integrity and translational fidelity [14,15,16,17]. These modifications play essential roles in diverse biological processes, including stem cell differentiation, stress adaptation [18], immune responses [19], and circadian rhythm regulation [20]. Disruption in the regulation of RNA modifications, often referred to as “epitranscriptomic dysregulation,” has been implicated in a growing number of diseases, including cancer [21,22], neurodevelopmental disorders [23], and metabolic syndromes [24], underscoring their importance in maintaining cellular homeostasis.

### 1.2. Pseudouridine: The Most Abundant RNA Modification, Catalyzed by Pseudouridine Synthases

Pseudouridine (Ψ) is the most abundant and one of the earliest discovered RNA modifications, found in a wide range of RNA species including tRNA, rRNA, snRNA, and mRNA [25,26]. It is formed by the isomerization of uridine, in which the uracil base is linked to the ribose via a carbon-carbon (C5–C1′) rather than a nitrogen-carbon (N1–C1′) glycosidic bond [26]. This seemingly subtle change significantly enhances the thermodynamic stability of RNA by improving base stacking and enabling additional hydrogen bonding, which can influence RNA structure and function. Studies have shown that abnormal Ψ levels are associated with clinical progression of cancer [27]. The conversion of uridine to Ψ is catalyzed by a family of enzymes known as pseudouridine synthases (PUS), which recognize specific sequences or structural elements in target RNAs [28,29]. These enzymes are evolutionarily conserved and function either as stand-alone proteins or as part of RNA-guided ribonucleoprotein complexes [26]. Pseudouridylation affects diverse cellular processes such as translation, RNA splicing, and stress responses, and its dynamic regulation in mRNA has expanded the understanding of its role from static structural maintenance to a key player in post-transcriptional gene regulation (Table 1).

### 1.3. The Pseudouridine Synthase Family: Spotlight on PUS7

The PUS family comprises a group of conserved enzymes responsible for catalyzing the site-specific isomerization of uridine to Ψ in various RNA molecules. In humans, the PUS family includes multiple members [17,26,43,44,45,46,47,48], such as PUS1, PUS3, PUS7, PUS10, TRUB1, TRUB2, PUSL1, PUS7L, DKC1, RPUSD1-4. PUS and associated enzymes act on diverse RNA substrates, including tRNA, rRNA, snRNA, and mRNAs, and are distributed across distinct cellular compartments such as the nucleus, cytoplasm, nucleolus, mitochondria, and Cajal bodies (Table 1). Their activities are often regulated by stress responses, nutrient availability. Developmental cues, and metabolic conditions, link pseudouridylation to cellular adaptation and disease. Overall, these enzymes highlight the functional diversity of pseudouridylation, spanning basic RNA structural stabilization, translation fidelity, mitochondrial homeostasis, stress responses, cell differentiation, immunity, and disease pathogenesis [26,49] (Table 1). Some function independently, while others operate as components of larger ribonucleoprotein complexes. Among these, PUS7 has emerged as a particularly versatile and dynamic enzyme. Initially identified for its role in modifying tRNAs and small nuclear RNAs, PUS7 has more recently been implicated in the pseudouridylation of mRNAs in a context-dependent manner, especially under stress conditions. PUS7 contains conserved catalytic domains characteristic of the TruD family of synthases and exhibits both nuclear and cytoplasmic localization [50,51,52]. Its regulated activity and ability to influence mRNA translation have linked it to key cellular processes such as cell proliferation, apoptosis, stem cell maintenance, stress adaptation, and tumor progression, making it a growing focus of investigation in RNA biology and disease research (Table 2 and Table 3).

### 1.4. PUS7 as a Disease-Linked RNA Modifier: Basis for Focus

PUS7 has emerged as a compelling focus in disease research due to its unique regulatory roles in RNA modification and its selective activity across cell types and stress conditions. PUS7 targets mRNAs and non-coding RNAs, influencing RNA stability, splicing, and translation, which are critical for maintaining cellular homeostasis [56]. Dysregulation of PUS7 has been implicated in various pathological contexts, including various types of cancer, where it contributes to oncogenic processes such as cell proliferation, metastasis, metabolic reprogramming, and evasion of apoptosis (Table 2 and Table 3). Notably, PUS7 interacts with protein partners such as HSP90, ANLN, and SIRT1, forming regulatory axes that further amplify its impact on disease progression [61,62,63]. Its context-specific expression and non-redundant function within the PUS family make PUS7 a promising candidate for targeted therapeutic intervention and a potential biomarker for disease prognosis (Table 2).

## 2. PUS7 Biology

### 2.1. Gene and Protein Structure

The human PUS7 gene is located on chromosome 7q22.1 and encodes PUS7, a member of the TruD family of PUS enzymes. The canonical PUS7 protein consists of a conserved TruD catalytic domain, which contains essential amino acid residues responsible for the isomerization of uridine to Ψ. This catalytic core is evolutionarily conserved from yeast to humans, reflecting its fundamental role in RNA modification. Additionally, PUS7 possesses an N-terminal extension that is thought to contribute to substrate recognition or subcellular localization [26]. The conservation of the catalytic domain across isoforms suggests that pseudouridylation activity is a shared core function. Further characterization of domain-specific roles and isoform-specific expression patterns may help clarify the diverse biological functions of PUS7 in both normal physiology and disease.

PUS7 is highly evolutionarily conserved across species, underscoring its fundamental role in RNA biology [60]. Homologs of PUS7 have been identified in organisms ranging from yeast (*Saccharomyces cerevisiae*) to humans, with significant sequence and structural conservation within the catalytic TruD domain responsible for pseudouridylation activity. In yeast, PUS7 (also called Pus7p) was initially characterized for its role in modifying specific uridines in tRNA and small nuclear RNA (snRNA), functions that are largely retained in higher eukaryotes. The preservation of key catalytic residues and structural motifs across diverse taxa suggests that the enzymatic mechanism and substrate recognition features of PUS7 are critical for cellular function. In both invertebrate and vertebrate models, PUS7 orthologs have been implicated in essential processes, highlighting a conserved biological relevance beyond basic RNA modification [52]. This evolutionary conservation not only reflects the essential nature of PUS7’s activity but also provides a valuable framework for studying its functions using model organisms.

### 2.2. Substrate Specificity and Activity

#### 2.2.1. Known RNA Substrates

PUS7 modifies a diverse array of RNA substrates, reflecting its multifaceted role in RNA metabolism (Table 2). Initially characterized by its activity on transfer RNA (tRNA), PUS7 catalyzes the pseudouridylation of specific uridine residues, such as position 13 in several tRNA species, which is critical for maintaining tRNA stability and proper folding. In small nuclear RNAs (snRNAs), PUS7 contributes to the modification of U2 snRNA, a central spliceosomal RNA, enhancing snRNA structure and function, and potentially influencing pre-mRNA splicing [25,72,73,74]. More recently, PUS7 has been shown to modify messenger RNA (mRNA) in a stress-responsive and cell-type-specific manner [18,40]. Pseudouridylation of mRNA by PUS7 can impact transcript stability, translation efficiency, and ribosome dynamics, often in response to environmental cues such as heat shock or nutrient deprivation [18,52]. These findings expand the functional scope of PUS7 beyond structural RNAs, positioning it as a regulator of gene expression at the post-transcriptional level. The broad substrate repertoire of PUS7 underscores its importance in maintaining RNA homeostasis and its potential to influence diverse biological pathways in both normal and disease context.

#### 2.2.2. Mechanisms of Site-Specific Pseudouridylation

PUS enzymes operate through two distinct mechanisms: the guide-independent and guide-dependent pathways [40,75]. In the guide-dependent pathway, pseudouridylation is carried out by a multi-protein ribonucleoprotein complex known as the H/ACA small nucleolar ribonucleoprotein (snoRNP). This complex includes a guide RNA (snoRNA) that base-pairs with the target RNA (substrate) to direct site-specific modification. The catalytic activity is mediated by the core enzyme dyskerin (DKC1), in cooperation with three essential protein components: NHP2, NOP10, and GAR1 [76,77]. Substrate recognition in this pathway relies on snoRNA scaffolds, enabling the modification of ribosomal RNAs (rRNAs) and small nuclear RNAs (snRNAs), processes essential for ribosome biogenesis and pre-mRNA splicing.

In contrast, the guide-independent pathway is mediated by stand-alone PUS enzymes that directly recognize specific uridine residues within their RNA substrates, based on local sequence motifs and structural features, without requiring guide RNAs. Site-specific pseudouridylation in this context is a tightly regulated process, ensuring precise modification of uridines across diverse RNA molecules. This specificity modification is exemplified by PUS7, a stand-alone PUS enzyme that directly recognizes sequence motifs such as UG Ψ AR (R = A/G) [18,60], and different locations within its RNA substrates. In tRNAs, for example, PUS7 targets conserved structural elements located in the terminal loop to catalyze modification at defined positions such as Ψ13, whereas U35 to Ψ35 conversion in U2 snRNA occurs in a single-stranded region linking two stem-loop structures [51]. Moreover, PUS7 can induce cell-type-specific tRNA pseudouridylation to regulate tumorigenesis [53]. A recent study using PUS7-knockout HeLa cells and the 2-bromoacrylamide-assisted cyclization sequencing (BACS) approach demonstrated that PUS7 activity occurs not only in cytoplasmic tRNAs but also in mitochondria, where it catalyzes the modification of Ψ50 in mt-tRNA^Met^ [30]. In addition to tRNA, PUS7-mediated Ψ modifications have also been reported in mRNAs [30,57]. The site-directed mutagenesis has shown that the PUS7 consensus Ψ site in the 3′ untranslated region of ATF4 mRNA is critical for the timely induction of ATF4 protein expression [18]. PUS7-mediated pseudouridylation appears to be dynamic and context-dependent, influenced by stress signals, cellular differentiation states, and interactions with RNA-binding proteins or cofactors [61,62,63] (Table 3).

Although the precise rules governing PUS7 substrate recognition in mRNAs remain incompletely understood, recent transcriptome-wide mapping techniques, such as PSI-seq [52,78,79], Nanopore direct RNA-seq [80,81], BACS [30], and bisulfite-induced deletion sequencing (BID-seq) [57,82,83,84], have revealed a set of reproducible pseudouridylation sites. These findings suggest that PUS members act through a combination of sequence preference and structural accessibility, enabling them to selectively regulate RNA fate and function in response to changing cellular conditions. A recent study demonstrated that knockdown of multiple PUS enzymes, including PUS7, followed by BID-seq revealed both distinct and overlapping Ψ sites across transcripts regulated by PUS enzymes [57]. PUS7 recognizes targets containing a UGΨAR motif, whereas TRUB1 modifies Ψ within a UG Ψ CN (N = A,C,G, or U). Some PUS enzymes, such as PUS1, PUS7, and TRUB1, act independently by directly recognizing and modifying their RNA substrates based on sequence or structural cues. By contrast DKC1 relies on H/ACA snoRNAs to direct site-specific pseudouridylation of rRNAs and snRNAs [26,31,41]. This division between independent and complex-dependent enzymes highlights the diverse mechanisms by which PUS family members regulate the Ψ landscape across different classes of RNA. Together, these two mechanistically distinct pseudouridylation pathways underscore the complexity and precision of RNA regulation through site-specific chemical modification.

### 2.3. Regulation of PUS7 Expression and Activity

The expression and activity of PUS7 are tightly regulated at multiple levels to ensure appropriate pseudouridylation in response to cellular context and environmental cues. Transcriptionally, PUS7 expression varies across tissues and developmental stages, with higher levels often observed in proliferative or stress-responsive cells. In pathological contexts such as cancer, PUS7 is frequently upregulated (Table 3), likely reflecting its responsiveness to oncogenic signaling pathways. However, a subset of cancer types exhibits reduced PUS7 expression (Table 3), which may result from tissue-specific regulatory mechanisms or reflect context-dependent roles, including a potential tumor-suppressive function [58,69]. These observations underscore the functional complexity of RNA-modifying enzymes like PUS7, whose biological impact is shaped by the molecular landscape of each tumor type. Elucidating the mechanisms underlying PUS7 downregulation in certain cancers may offer new insights into tumor heterogeneity and inform precision therapeutic strategies.

Stress conditions like heat shock, nutrient deprivation, and endoplasmic reticulum stress, have been shown to enhance PUS7-mediated pseudouridylation of mRNA, implying that its activity is dynamically regulated in response to environmental signals (Table 3). Pathogenic variants in the PUS7 gene cause a deficiency in RNA-independent pseudouridylation [85], which may contribute to tumorigenicity. Despite these insights, the precise molecular mechanisms governing PUS7 regulation remain incompletely understood, highlighting an important area for future investigation into how its dysregulation contributes to disease pathogenesis.

## 3. Functional Role of PUS7 in Cancer Cell Biology

### 3.1. Cell Proliferation and Survival

**Figure 1:**
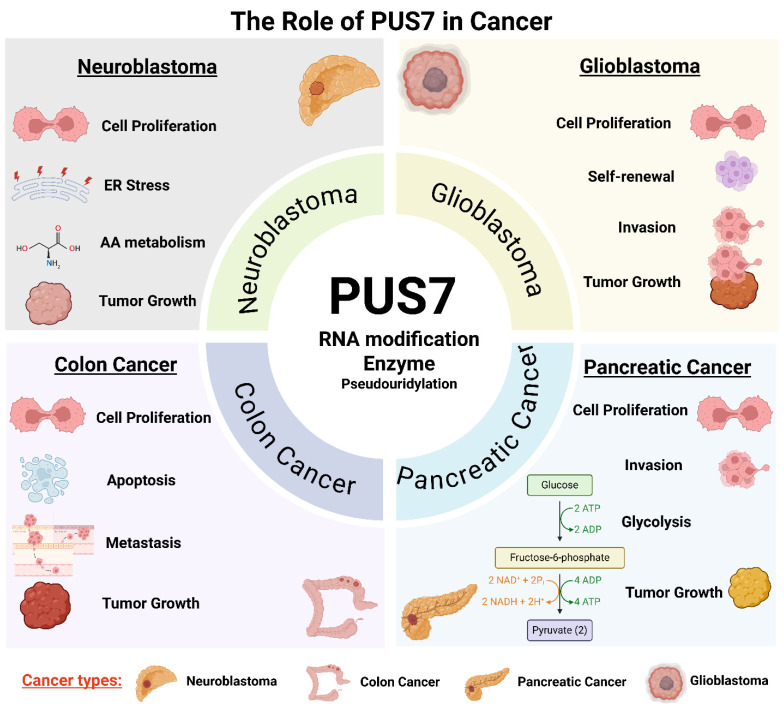
**The multifaceted role of PUS7 in cancer through distinct cellular and biological pathways.** PUS7, a stand-alone PUS, contributes to tumorigenesis and cancer progression through context-dependent mechanisms in diverse cancer types. PUS7 is implicated in regulating cell proliferation, invasion, stress responses, apoptosis evasion, and RNA metabolic rewiring. Across these contexts, PUS7 modifies key RNAs (tRNAs, mRNAs, and non-coding RNAs), altering transcript stability, translation efficiency, and cellular adaptability. This integrative figure highlights PUS7 as a central post-transcriptional regulator linking RNA modification to diverse oncogenic processes. Note: ER: endoplasmic reticulum; AA: amino acid. (Created with BioRender.com, accessed on 1 August 2025).

**Figure 2:**
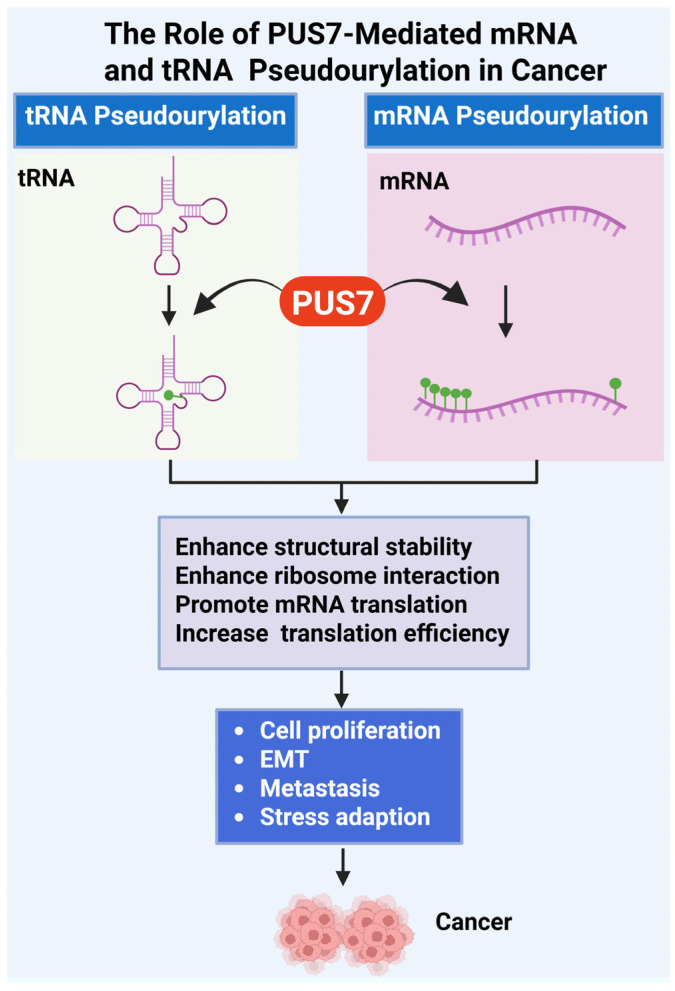
**Role of PUS7-Mediated tRNA and mRNA Pseudouridylation in Cancer Progression.** This figure illustrates the oncogenic mechanisms driven by PUS7, a pseudouridine synthase that catalyzes Ψ of both tRNAs and mRNAs [18,53]. PUS7-mediated pseudouridylation enhances the structural stability of RNA molecules, increases their affinity for ribosomal components, and promotes efficient ribosome assembly and translation initiation. Ψ modifications in tRNA and mRNA support cancer cell fitness by promoting abnormal cell proliferation, epithelial–mesenchymal transition (EMT), metastatic dissemination, and adaptation to stress conditions such as nutrient deprivation or oxidative damage. Dysregulated expression or activity of PUS7 therefore facilitates tumorigenesis through post-transcriptional reprogramming of gene expression. (Created with BioRender.com, accessed on 1 August 2025).

PUS7, a stand-alone PUS, plays a critical role in promoting cancer cell proliferation and survival through its RNA-modifying activity (Figure 1, Table 3). By catalyzing site-specific pseudouridylation on tRNAs, mRNAs, and non-coding RNAs, PUS7 regulates RNA stability, translation efficiency, and cellular stress responses, processes essential for sustaining rapid cancer cell growth. In neuroblastoma, for instance, PUS7-mediated pseudouridylation of regulatory mRNAs such as ATF4 enhances the translation of proteins involved in amino acid transport, redox balance, and the integrated stress response. These molecular effects support oncogene-driven survival programs, including MYC- and MYCN-mediated transcriptional networks [18]. Elevated PUS7 expression has been reported in multiple types of cancer, including pancreatic cancer, ovarian cancer, breast cancer, neuroblastoma, non-small cell lung cancer, hepatocellular carcinoma, colorectal cancer, and glioblastoma (Table 3). Genetic knockdown, knockout, or pharmacological inhibition of PUS7 reduces proliferation across various cancer cell types, such as pancreatic cancer [61], clear cell renal cell carcinoma [65], neuroblastoma [18], non-small cell lung cancer [70], colorectal cancer [62,64], and glioblastoma stem-like cells derived from diverse GBM subtypes [53], and leads to decreased tumor cell viability, increased apoptosis, and impaired tumor growth in vivo (Table 3, Figure 1). Moreover, gain-of-function studies demonstrate that PUS7 overexpression promotes cancer cell proliferation [57]. Collectively, these findings position PUS7 as a critical regulator of cancer cell homeostasis and a promising therapeutic target (Figure 2).

### 3.2. Cell Cycle and Apoptosis

PUS7 contributes to tumor progression by modulating key cellular processes such as the cell cycle and apoptosis. Through its pseudouridylation activity, PUS7 influences the expression and stability of RNAs encoding cell cycle regulators, enabling efficient progression through critical checkpoints. In the 6 types of cancer (bladder cancer, kidney-papillary cell carcinoma, lower grade-glioma, thyroid cancer, liver, and sarcoma), gene set enrichment analysis revealed that PUS7-high subset was enriched for G2/M checkpoint, mitotic spindle, PI3K/AKT/mTOR signaling pathways compared to the PUS7-low subsets. These pathways were associated with cell cycle progression across all 6 cancer types [86]. Functional studies have shown that PUS7 knockdown induces cell cycle arrest [18]. In contrast, PUS7 overexpression is associated with enhanced cyclin expression, accelerated cell proliferation, and reduced apoptosis [61]. Conversely, genetic or pharmacologic inhibition of PUS7 results in cell cycle arrest and reduced mitotic activity. Additionally, PUS7 plays a pro-survival role by stabilizing transcripts involved in anti-apoptotic signaling pathways; its depletion activates apoptotic cascades, including caspase cleavage and upregulation of pro-apoptotic genes [64]. These findings highlight PUS7 as a key epitranscriptomic regulator that maintains proliferative and survival advantages in cancer cells while suppressing terminal growth arrest programs (Figure 2, Table 3).

### 3.3. Invasion and Metastasis

PUS7 has emerged as a key regulator of cancer cell invasion and metastasis by modulating the post-transcriptional landscape of pro-metastatic genes. Through catalyzing site-specific pseudouridylation of mRNAs involved in epithelial–mesenchymal transition (EMT), cytoskeletal remodeling, and extracellular matrix (ECM) interactions, PUS7 enhances the stability and translational efficiency of transcripts that drive invasive behavior. Elevated PUS7 expression correlates with increased migratory capacity, matrix degradation, and upregulation of EMT markers such as N-cadherin and Vimentin. Conversely, inhibition or knockdown of PUS7 impairs these processes, resulting in reduced invasion and metastasis both in vitro [63,64,70] and in vivo [63].

In pancreatic cancer, PUS7 knockdown significantly suppressed the migratory and invasive abilities of pancreatic cancer cells, increased E-cadherin levels, and reduced V-cadherin and Vimentin expression. In contrast, PUS7 overexpression enhanced the migratory and invasive capacity of pancreatic cancer cells while inducing the opposite changes in EMT-associated protein levels [61]. Additionally, gain-of-function studies revealed that PUS7 overexpression promotes migration and invasion of colorectal cancer cells through upregulation of LASP1 [63]. Since transcription factors and signaling mediators such as ZEB1, SNAIL, E2F targets, and TGF-β effectors are known to orchestrate metastatic progression [87,88], it would be important to determine whether PUS7 regulates these effectors and contributes to cancer progression.

The role of PUS7 in metastasis was investigated using an in vivo colorectal cancer metastasis model [63]. Colorectal cancer cells with either PUS7 knockdown or PUS7 overexpression were injected into the tail veins of BALB/c nude mice. After eight weeks, histological analysis of lung tissues revealed a significant reduction in metastatic nodules in the PUS7 knockdown group, while PUS7 overexpression led to a marked increase in both the incidence and burden of lung metastases [63]. These results demonstrate that PUS7 promotes colorectal cancer metastasis in vivo. Collectively, these findings suggest that PUS7 reprograms RNA regulatory pathways to promote tumorigenesis by enhancing invasion and metastatic dissemination (Figure 2).

### 3.4. Stress Response and Chemoresistance

PUS7 plays a pivotal role in cellular stress adaptation and contributes to cancer progression. Through pseudouridylation of specific mRNAs and tRNAs, PUS7 modulates the translation of stress-responsive genes involved in amino acid metabolism, redox regulation, and the integrated stress response (ISR). For instance, PUS7 enhances translation of ATF4, a central ISR transcription factor, thereby promoting cellular adaptation to amino acid deprivation and endoplasmic reticulum stress conditions encountered in the MYCN-driven tumor microenvironment [18]. This adaptive advantage enables cancer cells to survive therapeutic insults. Additionally, PUS7 activity has been linked to the stabilization and efficient translation of mRNAs encoding drug efflux transporters and anti-apoptotic proteins, further contributing to resistance against chemotherapeutic agents. Inhibition or depletion of PUS7 sensitizes cancer cells to chemotherapy by impairing stress tolerance and promoting apoptosis, highlighting its potential as a target to overcome drug resistance (Figure 2).

### 3.5. The Impact of PUS7 on Signaling Pathways

Several studies have shown that PUS7 contributes to tumor progression by modulating key oncogenic signaling pathways. In colorectal cancer, PUS7 regulates the PI3K/Akt/mTOR signaling cascade, where PUS7 knockdown in colorectal cancer cells led to reduced phosphorylation of PI3K, Akt, and mTOR, while PUS7 overexpression enhanced their phosphorylation in colon cancer cells. Additionally, in colorectal cancer, PUS7 exerts oncogenic effects by interacting with Sirtuin 1 (SIRT1) and activating the Wnt/β-catenin signaling pathway [62]. In pancreatic cancer, PUS7 promotes malignant phenotypes through its interaction with anillin (ANLN), thereby activating the MYC signaling pathway. In neuroblastoma and lymphoma, MYCN and MYC function as upstream regulators of PUS7, driving its expression and contributing to tumor progression [18]. In GBM cells, PUS7 regulates GSC growth through a tyrosine kinase 2 (TYK2)-mediated interferon (IFN) pathway [53] (Table 3). Together, these findings underscore PUS7 as a key oncogenic regulator that promotes tumor progression by modulating multiple signaling pathways.

### 3.6. The Contribution of PUS7 to Tumor Development in Animal Models

PUS7 plays a critical role in promoting tumor growth and progression in multiple in vivo cancer models [18,53,61,62]. In immunodeficient NOD scid gamma (NSG) mice, transplantation of glioblastoma stem cells with knockdown of PUS7 significantly inhibited tumor progression and prolonged survival compared to controls [53]. In neuroblastoma, PUS7 overexpression markedly accelerated xenograft tumor growth and reduced the survival of tumor-bearing mice [18]. Similarly, in colorectal cancer, PUS7 knockdown suppressed tumorigenicity in vivo [62]. In pancreatic cancer, PUS7 knockdown significantly reduced tumor growth in mouse xenografts and was associated with a decrease in Ki-67–positive proliferating cells within tumor tissues [61] (Figure 1 and Figure 2).

Collectively, these in vivo studies demonstrate that PUS7 is a critical driver of tumor growth and progression across diverse cancer types, highlighting its potential as a therapeutic target in oncology.

## 4. PUS7 as a Therapeutic Target

### 4.1. Current Inhibitors or Approaches Targeting PUS7

Currently, efforts to directly target PUS7 are in the early stages, with only a few chemical inhibitors or approaches reported to date. One notable example of studies is using PUS7 inhibitors (PUS7i), including C4 and C17, which have been identified and shown to alter PUS7 enzymatic activity. Among them, C17 exhibited a stronger and dose-dependent inhibitory effect on patient-derived glioblastoma stem cells in a PUS7-dependent manner in vitro and that it preferentially targets glioblastoma stem cells compared to neural stem cells. Notably, C17 has been tested in animal models, where treatment significantly suppressed glioblastoma stem cells-derived tumor growth and prolonged survival in tumor-bearing NSG mice (Table 2). These effects were accompanied by reduced levels of Ψ in the treated tumors, indicating successful on-target activity [53]. However, the pharmacokinetic properties and downstream regulatory functions of PUS7 inhibitors remain to be fully characterized.

Another study investigated the use of PUS7i in a different cancer type, neuroblastoma. Functional assays have shown that targeting PUS7 impair cancer cell proliferation in neuroblastoma models where PUS7 is upregulated. Targeted inhibition of PUS7 abrogated MYCN-induced ATF4 expression and suppressed the production of amino acid transport proteins. Moreover, MYCN activation by 4-hydroxytamoxifen (4-OHT) promoted NB cell proliferation but concurrently sensitized cells to PUS7i treatment [18] (Table 2).

Beyond direct inhibition, alternative strategies to disrupt PUS7 function include RNA interference, CRISPR-mediated gene editing, and targeting upstream regulatory pathways that control its expression. Given the growing recognition of RNA-modifying enzymes as key regulators in cancer and other diseases, the development of selective, potent PUS7 inhibitors represents a promising therapeutic avenue. Nonetheless, further medicinal chemistry efforts, structure-based drug design, and in vivo validation will be critical to advancing these compounds toward clinical application.

### 4.2. Therapeutic Potential and Challenges

The therapeutic potential of targeting PUS7 lies in its emerging role as a regulator of RNA modifications that contribute to disease progression, particularly in cancers where they are aberrantly expressed or hyperactive. Inhibition of PUS7 has been shown to impair tumor cell proliferation and stress adaptation, making it an attractive candidate for novel epitranscriptomic-based therapies [18,53]. Moreover, because PUS7-mediated pseudouridylation selectively affects subsets of transcripts involved in translation, cell cycle regulation, and survival, its inhibition could provide a degree of specificity not easily achieved with broader gene expression modulators. However, several challenges remain. First, the lack of well-characterized, selective PUS7 inhibitors limits current therapeutic exploration. Second, because pseudouridylation is essential in normal cellular physiology, systemic inhibition may lead to off-target effects or toxicity. Furthermore, the context-dependent nature of PUS7 activity complicates the prediction of therapeutic responses across different tissues or tumor types. Addressing these challenges will require a deeper understanding of PUS7’s RNA targets, tissue-specific functions, and compensatory pathways, as well as the development of precise tools for modulating its activity in vivo.

### 4.3. Strategic Integration of Combination Therapies

Exploring combination therapy strategies involving PUS7 offers a promising avenue to enhance therapeutic efficacy while minimizing off-target effects. Given PUS7’s role in supporting stress tolerance and translational reprogramming in cancer cells, its inhibition may render tumors more vulnerable to additional insults, such as DNA damage, oxidative stress, or nutrient deprivation. This opens the door for combining PUS7 inhibitors with chemotherapy, radiation, or targeted agents that disrupt complementary survival pathways. For example, cancers with heightened dependence on the integrated stress response or aberrant RNA processing may exhibit synthetic lethality when PUS7 is suppressed. Additionally, combining PUS7 inhibition with agents targeting mRNA translation or epigenetic modifiers could synergistically impair tumor growth by disrupting layered regulatory networks. Importantly, these strategies could allow for lower dosing of each agent, potentially reducing toxicity. However, successful implementation will require systematic screening to identify actionable synthetic lethal partners and validation in disease-relevant in vivo models. Integrating PUS7 targeting into rational combination therapies represents a compelling strategy to exploit cancer-specific vulnerabilities rooted in RNA regulation.

### 4.4. Delivery Considerations for Targeting RNA-Modifying Enzymes

Effective delivery of therapeutics targeting RNA-modifying enzymes, such as PUS7, presents a significant challenge in the development of RNA epitranscriptomic-based therapies. Small-molecule inhibitors must achieve sufficient bioavailability, stability, and tissue-specific distribution to reach intracellular targets, particularly within the nucleus or cytoplasm where enzymes like PUS7 function. Additionally, given the broad expression of RNA-modifying enzymes in normal tissues, achieving selective targeting of diseased cells, such as cancerous or inflamed tissues, is critical to minimizing off-target effects and toxicity. Nanoparticle-based delivery systems, antibody-drug conjugates, and ligand-directed carriers are emerging strategies to enhance specificity and improve drug accumulation at disease sites. For genetic approaches, such as siRNA, shRNA, or CRISPR-based gene editing, efficient delivery systems must also protect nucleic acids from degradation and facilitate uptake into target cells. Crossing biological barriers, such as the blood–brain barrier in neurological disorders, adds further complexity. As the therapeutic interest in RNA-modifying enzymes grows, advancing delivery technologies tailored for these intracellular targets will be essential to translate molecular insights into safe and effective clinical treatments.

## 5. Future Directions

### 5.1. What Regulates PUS7 Activity and Localization?

Despite growing interest in PUS7 as a key RNA-modifying enzyme, the upstream mechanisms that regulate its enzymatic activity and subcellular localization remain poorly understood. While PUS7 contains conserved domains critical for its catalytic function, little is known about how its activity is modulated in response to cellular signals such as stress, differentiation, or oncogenic transformation. Post-translational modifications, such as phosphorylation or ubiquitination, may alter PUS7’s activity, stability, or interactions with RNA substrates and protein cofactors, but these modifications have yet to be systematically characterized. Additionally, PUS7 exhibits both nuclear and cytoplasmic localization, suggesting it may shuttle between compartments in a context-dependent manner to access different RNA pools; however, the molecular cues governing this trafficking are still unknown. Notably, while the role of PUS7 mutations in cancer is largely unexplored, mutations in PUS7 have been reported to cause intellectual disability with growth retardation [89], accompanied by reduced Ψ13 levels in tRNAs. These observations indicate that PUS7 variants impair pseudouridylation and highlight the enzyme’s essential role in proper neuronal development and function [36]. Identifying the signaling pathways, binding partners, or environmental triggers that control PUS7’s distribution and function will be essential for understanding how pseudouridylation is dynamically regulated in health and disease. Future studies using live-cell imaging, mass spectrometry, and conditional knockout models will be critical to dissect the spatial and temporal control of PUS7 and its integration into broader gene regulatory networks.

### 5.2. Unidentified RNA Targets in Disease Contexts

Although several RNA substrates of PUS7 have been identified, including specific tRNAs, snRNAs, and stress-responsive mRNAs, a substantial portion of its target repertoire in disease contexts remains unknown. Emerging evidence suggests that PUS7 may modify distinct sets of tRNA and mRNAs in a cell type- and condition-specific manner, particularly in diseases such as cancer [53]. However, the full spectrum of these RNA targets, and how their pseudouridylation impacts transcript stability, translation, or splicing, is still poorly characterized. Notably, disease-associated changes in RNA structure, expression levels, or RNA-binding protein interactions could alter PUS7 substrate specificity, introducing new or aberrant targets. Identifying and functionally validating these context-dependent RNA targets will be crucial to understanding the pathogenic roles of PUS7 and uncovering potential biomarkers or therapeutic vulnerabilities associated with its activity in human disease.

Although research has begun to uncover the role of pseudouridylation and PUSs in gynecological disorders [90,91], particularly the involvement of PUS7 [57,66,71], our understanding remains limited. Most current studies are preliminary, and the specific molecular mechanisms by which pseudouridylation contribute to the onset and progression of gynecological diseases, such as endometriosis, uterine fibroids, and uterine cancers, are still not fully elucidated. Moreover, comprehensive profiling of PUS enzyme expression, target RNAs, and functional consequences in relevant gynecological tissues is lacking. Further in-depth studies are needed to define the precise role of individual PUS enzymes in disease-specific contexts and to evaluate their potential as diagnostic biomarkers or therapeutic targets.

### 5.3. Role of PUS7 in RNA Epitranscriptomics and Cellular Plasticity

PUS7 plays an increasingly recognized role in the dynamic regulation of gene expression through chemical modifications of RNA. By catalyzing the pseudouridylation of tRNA, snRNA, and stress-responsive mRNA transcripts, PUS7 contributes to the functional diversity and regulatory potential of the transcriptome without altering the underlying nucleotide sequence. Pseudouridylation of mRNA by PUS7 can modulate RNA stability, translation efficiency, and ribosome engagement, thereby influencing the cellular proteome in a context-dependent manner. This capacity to reprogram translation in response to stress, differentiation cues, or oncogenic signals endows cells with greater plasticity, the ability to rapidly adapt to changing environments or functional demands [18,60]. In stem cells, PUS7 supports the maintenance of pluripotency by regulating translational programs critical for self-renewal [53]. In cancer, its dysregulated activity has been linked to the promotion of malignant phenotypes such as abnormal cell proliferation, resistance to stress, and therapeutic evasion. As a key enzyme in the epitranscriptomic network, PUS7 serves as a molecular switch that connects RNA modification to dynamic shifts in cell state and identity.

### 5.4. Unraveling the Overlapping Functions of PUS in RNA Biology

PUS constitutes a diverse family of 13 putative enzymes responsible for catalyzing the isomerization of uridine to Ψ in various RNA species, including tRNAs, rRNAs, snRNAs, and mRNAs [32] (Table 1). While individual PUS enzymes often exhibit substrate preferences, many have overlapping targets, leading to functional redundancy that complicates the elucidation of enzyme-specific roles. For example, some Ψ sites in HeLa transcripts are regulated by multiple PUS enzymes, as shown by knockdown of eight PUS enzymes, including PUS7, followed by identification using BID-seq [82]. This redundancy can mask the contribution of single enzymes to RNA modification, making it difficult to link specific pseudouridylation events to distinct cellular outcomes. Furthermore, the interplay between independent PUS enzymes and those operating within ribonucleoprotein complexes adds an additional layer of regulatory complexity. Understanding how these enzymes cooperate, compensate, or compete on shared RNA substrates is critical for deciphering the functional landscape of RNA pseudouridylation. Investigating these overlapping functions will provide insights into the regulation of RNA stability, translation, and processing, and may reveal novel mechanisms by which dysregulation of PUS enzymes contributes to disease, including cancer and gynecological disorders.

### 5.5. Importance of in Vivo Models and Clinical Validation

Despite growing evidence connecting PUS7 to disease-related processes in vitro, there remains a critical need for in vivo models and clinical validation to fully elucidate its physiological and pathological roles. Although a few studies have demonstrated the importance of PUS7 in vivo [18,53,61,63], the majority of current knowledge is derived from cell-based systems. While informative, these systems cannot fully recapitulate the complexity of tissue-specific regulation, immune interactions, and systemic physiological responses. The generation of conditional or tissue-specific PUS7 knockout mouse models would enable detailed dissection of its roles in development, homeostasis, and disease progression, including tumorigenesis and neurodevelopmental disorders. Furthermore, in vivo models are crucial for assessing the systemic effects and potential toxicities associated with therapeutic targeting of PUS7. On the clinical front, large-scale analyses of PUS7 expression and pseudouridylation signatures across patient cohorts are needed to evaluate its potential as a diagnostic biomarker or therapeutic target. Integrating transcriptome-wide Ψ mapping with clinical outcome data could further illuminate the prognostic significance of PUS7 activity. Together, comprehensive in vivo studies and robust clinical validation are essential for translating basic mechanistic insights into meaningful advances in disease diagnosis and treatment.

### 5.6. Ψ as a Potential Biomarker

Ψ, the most abundant RNA modification, has emerged as a promising biomarker candidate for cancer detection and monitoring. As a stable, non-degradable modified nucleoside, Ψ accumulates in biological fluids such as serum, plasma, and urine, particularly under conditions of elevated RNA turnover, a hallmark of many malignancies [92,93]. Elevated levels of circulating Ψ have been reported in various cancers, including lung, breast, prostate cancer, and hematologic malignancies, and often correlate with tumor burden or progression [93,94,95,96,97,98,99]. Its accumulation reflects enhanced transcriptional and translational activity in rapidly proliferating cancer cells, making it a potential indicator of disease state or therapeutic response [94]. Although Ψ lacks disease specificity and has yet to be implemented in clinical practice, recent advances in mass spectrometry and nucleoside profiling have enabled its inclusion in experimental liquid biopsy platforms [27,93,94,96,97,98,99,100,101,102,103]. To improve its clinical applicability, development and standardization of targeted assays with enhanced sensitivity and specificity are needed. Integrating Ψ detection with multi-omics approaches, such as metabolomics and transcriptomics, could improve its diagnostic and predictive value. Further validation in large, well-characterized cohorts is required to establish Ψ, particularly in relation to Ψ synthases like PUS7, as a robust, non-invasive cancer biomarker.

## 6. Conclusions

There is a growing interest in RNA-modifying enzymes as critical regulators of gene expression and emerging contributors to human disease. These enzymes, which catalyze a wide range of chemical modifications on tRNA, rRNA, mRNA, and non-coding RNAs, add a dynamic and reversible layer of regulation to the transcriptome. Recent studies have revealed that aberrant RNA modifications can profoundly influence RNA stability, translation, splicing, and localization, thereby contributing to the development and progression of cancer, neurological disorders, metabolic syndromes, and immune-related diseases. Enzymes such as PUS, methyltransferases, and demethylases, among others, are now recognized not only for their physiological roles but also as potential biomarkers and druggable targets. This expanding field highlights the importance of understanding RNA modifications in disease contexts and opens new avenues for therapeutic intervention aimed at restoring RNA regulatory balance.

PUS7 represents a promising molecular node that links RNA modification to diverse disease phenotypes through its context-specific pseudouridylation activity. By modifying select mRNAs, tRNAs, and non-coding RNAs, PUS7 influences RNA stability, localization, and translational efficiency, thereby exerting broad regulatory control over gene expression programs. Emerging evidence suggests that aberrant PUS7 activity contributes to disease states such as cancer, where it promotes oncogenic pathways including MYC/MYCN signaling, metabolic reprogramming, and cell proliferation. Its integration into regulatory networks/pathways and interactions with key protein cofactors further underscore its central role in pathophysiological processes. As a modulator of RNA epitranscriptomic landscapes, PUS7 serves as a critical interface between RNA-level regulation and downstream cellular phenotypes, highlighting its potential as a diagnostic marker and therapeutic target in RNA-driven diseases.

## Figures and Tables

**Table 1:** Key Features of Major Pseudouridylases.

Enzyme	Known RNA Targets	Cellular Localization	Regulatory Conditions	Associated Functions	Ref.
PUS1	tRNA, snRNA, mRNA	Nucleus, Mitochondria	Stress response, metabolic cues	Mitochondrial translation, RNA stability, splicing regulation	[4,26,30,31,32,33]
PUS3	tRNA (anticodon stem–loop)	Nucleus, Cytoplasm	Developmental regulation, neuronal contexts	Essential for tRNA modification; mutations linked to intellectual disability and neurodevelopmental disorders	[31,34,35]
PUS7	tRNA, snRNA, mRNA	Nucleus, Cytoplasm	Nutrient availability, stress signals	Translational control, stem cell differentiation, stress adaptation, splicing regulation, neurodevelopmental disorder	[30,31,32,36]
PUS7L	mRNA	Nucleus	Not well characterized	RNA pseudouridylation in transcripts	[37]
PUSL1	Mitochondrial tRNA	Mitochondria	Oxidative stress, mitochondrial function	Regulation of mitochondrial translation and RNA modification	[26,37]
PUS10	tRNA (Ψ at position 54, 55), snRNA	Cytoplasm, Nucleus, and Mitochondria	Apoptotic signals, immune activation	Apoptosis regulation, innate immune function	[26,31]
TRUB1	tRNA (Ψ55), mRNA	Nucleus, Cytoplasm	Growth conditions, differentiation cues	Translation fidelity, RNA structural stability	[26,30,38]
TRUB2	tRNA (mitochondrial), mRNA	Mitochondria	Mitochondrial stress, oxidative stress	Mitochondrial translation, respiratory function	[26,32,39]
DKC1	rRNA (28S, 18S), snRNA (H/ACA snoRNP-associated)	Nucleolus, Cajal bodies	Cell cycle, telomere maintenance	Ribosome biogenesis, telomerase RNA stabilization, splicing regulation, DKC-1 mutation related diseases	[26,31,40,41]
RPUSD1	rRNAs (mitochondrial), mRNA	Mitochondria, Cytoplasm	Energy demand and metabolic regulation	Maintains mitochondrial ribosome function and respiratory capacity, stabilize elF4E mRNA via its RluA domain	[42]
RPUSD2	rRNA, mRNA	Nucleolus, Mitochondria	Developmental regulation	Ribosome assembly, mitochondrial gene expression	[26,31]
RPUSD3	rRNAs, mRNA (mitochondrial)	Mitochondria	OXPHOS regulation	Contributes to mitochondrial ribosome biogenesis and translation	[26,31,32,39]
RPUSD4	rRNAs (mitochondrial)	Mitochondria, Nucleus	Metabolic stress, hypoxia	Ensures proper mitochondrial ribosome assembly and function, splicing regulation	[26,31,32]

**Table 2:** PUS7-Mediated Mechanisms in Disease: Functional Roles, Regulation, and Therapeutic Potential.

Aspect	Description	Biological Context (Cancer/Model)	Ref.
Functional Role			
Pseudouridylation of tRNAs	Modifies specific uridines to stabilize tRNA structure and maintain translation fidelity	General cellular function; disrupted in cancer	[53,54,55]
Pseudouridylation of mRNAs	Modifies specific uridines on mRNA	Dynamic modification affects pre-mRNA splicing efficiency, mRNA stability, translation efficiency, and stress response	[56,57,58,59]
Regulation of translational reprogramming and efficiency	Influences selective translation of stress- and proliferation-related transcripts	Promote cancer progress across multiple cancer types	[18,53,54,58]
Regulatory Role			
Expression regulation	Transcriptionally upregulated in proliferative, metastasis, and stress contexts	Pre-mRNA splicing, RNA stability, Translation	[52,59]
Impact on stem cell maintenance	Supports pluripotency via modulation of translation	Developmental biology, cancer stem cells, self-renewal	[53,54]
Subcellular localization	Shuttles between nucleus and cytoplasm for substrate access	Cellular stress that triggers cytoplasmic relocation	[60]
Interaction with cofactors	Bind with cofactors, such as SIRT1, HSP90, and ANLN	Modulation of substrate specificity and the functions of downstream regulatory effectors	[61,62,63]
Response to environmental stimuli	Alter the PUS7-mediated Ψ modifications	Modulation of transcript stability of stress-response genes to promote stress adaption	[18,52]
Pathways	Wnt/β-catenin, PI3K/AKT/mTOR, IFN pathway, MYCN/MYC	Promoting cancer progression in NB, PC, GSC, and CRC	[18,53,61,62,64]
Cellular processes	Cell proliferation, glycolysis, invasion, apoptosis	Promoting cancer progression across multiple cancer types	[18,53,61,62]
Therapeutic implications			
Target for small-molecule inhibition	Preclinical PUS7 inhibitors	Suppressing cancer cell proliferation in NB and GBM cell-based models as well as in a GBM xenograft model	[18,53]
Biomarker potential	The amount of secreted Ψ correlates with upregulation of PUS members, including PUS7	Predictive and diagnosis value	[65]

Note: PC: Pancreatic cancer; GBM: Glioblastoma; CRC: colorectal cancer; NB: Neuroblastoma; GSC: Glioblastoma stem cell; CRC: colorectal cancer.

**Table 3:** Summary of PUS7 Expression, Regulation, and Functional Insights in Cancer.

Diseases	Human Sample Types	Up/Down	Main Approaches	Contexts	Time	Ref.
Pancreatic cancer	PC tissues and cells	Increase in PC	Cellular, molecular, and animal model	Accelerates proliferation, motility, and glycolysis, and inhibits apoptosis through interaction with ANLN; promotes PC progression by activating the MYC pathway	2025 Jul	[61]
Ovarian cancer	OC tissues	Increase in OC	Transcriptome and qPCR	Prognostic signature	2025 May	[66]
Renal cell carcinoma	RCC tumors	Increase in RCC	TCGA, GEO, and GENT2 databases and bioinformatic analysis	Correlated with proliferation, prognosis markers, and overall survival	2025 Apr	[67]
Bladder cancer	BC tissues	Increased in BC	TCGA database and bioinformatic analysis	Poor survival outcome, positively correlated with Th2 infiltration, negatively associated with pDC and NK cell infiltration	2025 Apr	[68]
Neuroblastoma	NB tissue and cells	Increased in NB tissues and MYCN-overexpressed NB cells	Functional assays, xenograft model, ChIP-qPCR, microarray, proteomics, Nanopore direct RNA-seq, amino acid deprivation,	PUS7 is regulated by MYCN and is involved in NB proliferation, tumorigenicity, amino acid biosynthesis and transport, and is associated with poor prognosis in NB; the PUS7 inhibitor suppresses tumor growth	2024 Dec	[18,59]
Lymphoma	Burkitt Lymphoma cell line	Increased in lymphoma cell line	Burkitt lymphoma model cell line with Inducible MYC expression	MYC upregulates PUS7	2024 Dec	[18]
Papillary thyroid carcinoma	Metastatic and non-metastatic PTC	Decreased in PTC	Small RNA Ψ modification microarray (TCGA, GEO database), qPCR	miR-8082, tumor progression and metastasis	2024 Oct	[69]
Cervical carcinoma	HeLa cells	ND	PUS7 KO, BACS	Ψ changes in tRNA and mRNA	2024 Sep	[30]
Gastric cancer	GC tissues	Decreased in GC tissues	Ψ detection assay, polysome profiling assays, xenograft model	Inhibits gastric cancer (GC) cell proliferation and tumor growth, and enhances the translation efficiency of ALKBH3 through pseudouridylation	2024 Aug	[58]
Non-small cell lung cancer	NSCLC tissues and cell lines	Increased in NSCLC tissues and cell lines	qPCR, immunoblot, CCK8, migration and invasion assay, flow cytometry, IHC	Promotes NSCLC cell proliferation, migration, and invasion; associated with poor prognosis	2023 Jul	[70]
Clear cell renal cell carcinoma	VPR ccRCC cell lines	Increased in murine ccRCC model	VPR: murine model with the tubule-specific deletion of Vhl, Trp53, and Rb1, LC-MS, RNA-seq	Higher expression and more Ψ nucleosides excreted in VPR cells	2023 Apr	[65]
Cervical carcinoma	HeLa cells	ND	PUS7 KD, BID-seq	Identifies PUS7-mediated pseudouridine sites on mRNA	2023 Mar	[57]
Colorectal cancer	CRC tissues and cells	Increased in CRC tissues and cell lines	IHC, CCK-8 assay, KD experiments, Co-IP, xenograft	Promotes cell proliferation by interacting with SIRT1 and activating the Wnt/β-catenin signaling pathway	2023 Feb	[62]
Hepatocellular carcinoma	HCC tissues and cell lines	Increased in HCC tissues	Bioinformatic analysis of GSCA, q-PCR	Upregulation of PUS7 associated with poor survival	2022 Nov	[37]
Colon cancer	CC tissue and cell line	Increased in CC tissue and cell lines	Cell proliferation and invasion assays, Functional assays of PUS7 KD and overexpression	Promotes proliferation and invasion and suppresses apoptosis of colon cancer cells by activating the PI3K/AKT/mTOR signaling pathway; associated with poor survival rates.	2022 Apr	[64]
Ovarian cancer	OC tissues	Increased in OC tissues	Bioinformatics analysis from TCGA and GEO, IHC staining and tissue array	Potential diagnostic marker and therapeutic target	2021 Nov	[71]
Glioblastoma	GBM and GSC	Increased in GBM	GBM GlioVis portal analysis, IHC, cellular function assays, GSC transplantation and inhibitor treatment, LC-MS, Small RNA DM-Ψ-seq, transcriptome-wide Ψ sequencing	Regulates GSC growth, self-renewal, tRNA pseudouridylation, and translation; the PUS7 inhibitor suppresses tumor growth	2021 Sep	[53]
Colorectal cancer	CRC tissues and cells	Increased in CRC	Functional assay of PUS7 KD, migration and invasion assays, in vivo metastatic model, Co-IP RNA-seq and proteome profiling	Promotes CRC cell metastasis in vitro and in vivo; activation of the HSP90/PUS7/LASP1 axis is associated with poor prognosis	2021 May	[63]

Note: TMA: tissue microarray; OC: ovarian cancer; BC: Bladder cancer; PC: Pancreatic cancer; PTC: papillary thyroid carcinoma; NB: neuroblastoma; GC: Gastric cancer; NSCLC: non-small cell lung cancer; ccRCC: Clear cell renal cell carcinoma; HCC: Hepatocellular carcinoma; GBM; glioblastoma; GSC: glioblastoma stem cell; HPA: Human Protein Atlas; CRC: colorectal cancer; KD: knockdown; BACS: 2-bromoacrylamide-assisted cyclization sequencing; ND: not determined. The database’ URLs: TCGA: https://www.cancer.gov/tcga, (accessed on 1 August 2025) GlioVis portal: http://gliovis.bioinfo.cnio.es, (accessed on 1 August 2025) GEO: https://www.ncbi.nlm.nih.gov/geo/, (accessed on 1 August 2025) GENT2: http://gent2.appex.kr/gent2/ (accessed on 1 August 2025).

## Data Availability

No new data were created or analyzed in this study.
